# The role of promoter *cis*-element, mRNA capping, and ROS in the repression and salt-inducible expression of *AtSOT12* in *Arabidopsis*

**DOI:** 10.3389/fpls.2015.00974

**Published:** 2015-11-06

**Authors:** Jinhua Chen, Bangshing Wang, Jung-Sung Chung, Haoxi Chai, Chunlin Liu, Ying Ruan, Huazhong Shi

**Affiliations:** ^1^Pre-National Laboratory for Crop Germplasm Innovation and Resource Utilization, Hunan Agricultural UniversityChangsha, China; ^2^Department of Chemistry and Biochemistry, Texas Tech University, LubbockTX, USA

**Keywords:** AtSOT12, salt stress, gene regulation, ROS, promoter analysis, mRNA capping, polyadenylation

## Abstract

Inducible gene expression is a gene regulatory mechanism central to plant response to environmental cues. The inducible genes are often repressed under normal growth conditions while their expression levels are significantly elevated by conditions such as abiotic stresses. Induction of gene expression requires both *cis*-acting DNA elements and *trans*-acting proteins that are modulated through signal transduction pathways. Here we report several molecular events that affect salt induced expression of the *Arabidopsis AtSOT12* gene. Promoter deletion analysis revealed that DNA elements residing in the 5′ UTR are required for the salt induced expression of *AtSOT12*. Cytosine methylation in the promoter was low and salt stress slightly increased the DNA methylation level, suggesting that DNA methylation may not contribute to *AtSOT12* gene repression. Co-transcriptional processing of *AtSOT12* mRNA including capping and polyadenylation site selection was also affected by salt stress. The percentage of capped mRNA increased by salt treatment, and the polyadenylation sites were significantly different before and after exposure to salt stress. The expression level of *AtSOT12* under normal growth conditions was markedly higher in the *oxi1* mutant defective of reactive oxygen species (ROS) signaling than in the wild type. Moreover, *AtSOT12* transcript level was elevated by treatments with DPI and DMTU, two chemicals preventing ROS accumulation. These results suggest that repression of *AtSOT12* expression may require physiological level of ROS and ROS signaling.

## Introduction

Gene regulation is a fundamental molecular process governing growth, development and environmental response in all living organisms. In eukaryotes, gene expression can be regulated at almost all the steps from chromatin remodeling and transcription to post-transcription, protein translation and protein degradation. DNA methylation and histone modifications are major chromatin regulation mechanisms resulting in alternation of binding affinity of the DNA elements with their recognition protein factors. These epigenetic regulations have been implicated in human health and diseases (Hamidi et al., 2015; Seidel et al., 2015). In plants, epigenetic control of developmental processes such as embryogenesis and flowering has been well documented (Ahmad et al., 2010). Epigenetic regulation is also an important regulatory node for plant stress response (Chinnusamy and Zhu, 2009; Kim et al., 2015). For example, positive correlation between drought stress intensity and H3K9 histone acetylation of drought inducible genes was observed (Kim et al., 2008); and histone methylation at H3K4, a gene activation marker was also enriched in the drought stress upregulated genes (Ding et al., 2011, 2012; Kim et al., 2012). Implication of epigenetic regulation in salt stress response stemmed from the observation that the *Arabidopsis* mutants defective in histone acetylation and deacetylation enzymes showed hypersensitivity to salt (Chen and Wu, 2010; Kaldis et al., 2011; Luo et al., 2012). Moreover, Sani et al. (2013) showed that the H3K27me3, a histone modification resulting in gene repression, around the *HKT1* gene was decreased along with salt treatment. *HKT1* gene is an important salt tolerance determinant in plants (Zhang and Shi, 2013), and the decrease of histone methylation at H3K27 may lead to a rapid increase in *HKT1* gene transcription. Besides, histone modification has been implicated in inducible gene expression in response to other stress conditions such as cold and heat (Song et al., 2012; Liu et al., 2015). Although DNA methylation was also shown to be altered by stress treatments, the role of DNA methylation in abiotic stress response is still poorly understood (Kim et al., 2015).

Gene expression control at the transcription level is the most studied gene regulation mechanism. By studying abiotic stress induced or associated transcription factors and the promoters of the stress inducible genes, several important transcriptional regulatory networks have been elucidated in plants. The osmotic stress responsive genes are regulated by ABA-dependent and ABA-independent signaling pathways involving transcription factors and their recognition promoter *cis*-acting elements (Yoshida et al., 2014). The ABA-dependent pathway consists of the *cis*-acting element ABA-responsive element (ABRE) and the ABRE binding factors (ABFs), while the ABA-independent pathway includes dehydration-responsive element/C-repeat (DRE/CRT) and the DRE/CRT binding protein 2 (DREB2). Cold stress activates the ICE-CBF/DREB1-CRT/DRE pathway that upregulates cold responsive (COR) genes (Miura and Furumoto, 2013). The ICE1 (inducer of CBF expression 1) is a MYC-type transcription factor that binds to the promoter of CBF3 thus promote the production of this transcription factor (Chinnusamy et al., 2003). The CBF3 binds with the CRT/DRE *cis*-elements residing in the promoters of the COR genes and promotes the transcription of the COR genes (Maruyama et al., 2004). The activation of HSFs (heat stress transcription factors) and its binding with the *cis*-element HSEs (heat shock elements) in the promoters of HSPs (heat shock proteins) is another example of transcriptional regulation of gene expression in response to heat stress in plants (von Koskull-Döring et al., 2007). Besides transcriptional regulation, post-transcriptional regulation of gene expression in response to abiotic stress has also been considered as important regulatory steps (Mazzucotelli et al., 2008). Alternative splicing has been shown to play crucial roles in plant adaptation to environmental stresses (Filichkin et al., 2015), and increasing number of studies have backed up this notion since the application of RNA-seq in genome-wide transcriptome analysis. In contrast, involvement of the co-transcriptional mRNA capping and polyadenylation processes in plant stress response has been rarely studied. Jiang et al. (2013) reported that the *Arabidopsis* KH domain containing protein SHI1 and its interacting protein FRY2 are involved in repression of stress-inducible gene expression by modulating mRNA capping and polyadenylation site selection. The *Arabidopsis* Cleavage and Polyadenylation Specificity Factor 30 (CPSF30) is a key polyadenylation factor, and CPSF30 has been implicated in oxidative stress and defense response (Chakrabarti and Hunt, 2015). Overall, the role of mRNA capping and polyadenylation regulation in stress response is still largely obscure.

Salt stress induces a number of gene expression in plants. The majority of the salt inducible genes are also induced by osmotic stress treatment, but some salt inducible genes are not shared with osmotic stress (Kreps et al., 2002), suggesting that salt stress imposes plants with not only osmotic stress component but also ion toxicity that is specific to salt stress. The *Arabidopsis* sulfotransferase gene *AtSOT12* is one of the salt inducible genes identified as NaCl specific in the transcriptome analysis by Kreps et al. (2002). However, our further study on the functions of AtSOT12 revealed that *AtSOT12* gene is induced by multiple stress conditions including salt and osmotic stress (Baek et al., 2010; Chen et al., 2015). This suggests that the induction of *AtSOT12* expression by NaCl treatment may consist of both osmotic and ion stress components. In this study, we focus on the regulation mechanism of *AtSOT12* gene expression. We found that the *cis*-element responsible for salt inducible expression of *AtSOT12* gene resides at the 3′ end of the 5′-UTR. Salt stress increases *AtSOT12* mRNA capping and changes the polyadenylation site selection. Furthermore, *AtSOT12* gene repression requires reactive oxygen species (ROS) accumulation and signaling. These results provide novel mechanisms for salt responsive gene regulation.

## Materials and Methods

### Plant Materials and Growth Conditions

*Arabidopsis thaliana* Columbia-0 (Col-0), *Landsberg erecta* (Ler), and the *oxi1* mutant seeds were obtained from the *Arabidopsis* Biological Resource Center (ABRC). Seeds were surface sterilized by incubating in 20% Bleach/Clorox with 0.05% Triton X-100 for 15 min with shaking, followed by washing for five times with sterile water. Sterilized seeds were then suspended with 0.1% low melting point agarose (Sigma), and incubated at 4°C for 2–3 days. Seeds were planted on ½ MS agar medium (½ MS salts, 1.5% sucrose, 0.7% agars, pH 5.7). The plates were placed in a growth room at 22°C under a daily cycle of 16 h light and 8 h dark.

### Promoter Deletion Constructs

The promoter containing approximately 1 kb sequence upstream of the *AtSOT12* coding region, and promoter deletions from 5′ or 3′ end were PCR-amplified from genomic DNA by using the primers listed in Supplemental Table S1. The PCR fragments were inserted into the plasmid vector pCAMBIA 1318Z–LUC (Jiang et al., 2013) to create transcriptional fusion of *AtSOT12* promoter and the luciferase reporter gene. The resulting constructs were introduced into the *Agrobacterium* strain GV3101, and *Arabidopsis thaliana* Col-0 wild type plants were transformed with these constructs using *Agrobacterium*-mediated floral dip method (Clough and Bent, 1998).

### Luciferase Assay

Luciferase assay were performed by using T2 transgenic plants harboring the promoter deletion constructs. For luminescence imaging, 10-day-old seedlings growing on ½ MS agar plates were treated with 200 mM NaCl for 5 h, then sprayed uniformly with 1 mM luciferin in 0.01% Triton X-100 and kept in dark for 5 min before imaging. All images were acquired using a thermoelectrically cooled CCD camera (DU434-BV, Andor Technology, Connecticut). Quantification of luminescence intensity of the individual seedlings was carried out by using the Andor software provided by the camera manufacturer.

### Northern Blotting Analysis

Total RNA isolation was carried out according to Shi and Bressan (2006). Northern blotting was performed essentially following Chung et al. (2008). For promoter-LUC analysis, 10-day-old T2 seedlings with the promoter deletion constructs with or without NaCl treatment were collected for RNA extraction and Northern blotting. For *AtSOT12* gene expression analysis, 10-day-old seedlings of wild type and *abi1, abi2, abi3, sos1, sos2, sos3, oxi1* mutants with different treatments indicated in **Figure 5** were collected for RNA isolation and Northern blotting. The DNA probes for Northern blotting were PCR-amplified by using the following primers: *LUC*, tggagagcaactgcataagg, and tgacgcaggcagttctatgc; *AtSOT12*, atgtcatcatcatcatcagttcctg, and tcaagaagaaaatttaagaccagaacc; and β*-tubulin* gene (AT5G23860.1), cgtggatcacagcaatacagagcc, and cctcctgcacttccacttcgtcttc. The DNA probes were labeled with ^32^P-dCTP by using the Primer-It II Random Primer Labeling Kit (Stratagene).

### Promoter-GUS Analysis

The ∼1 kb full promoter sequence of *AtSOT12* gene was PCR-amplified and inserted in front of the GUS reporter gene in the plasmid vector pCAMBIA 1381Z. The resulting construct was transformed into *Arabidopsis thaliana* Col-0 wild type plants by using the *Agrobacterium*-mediated floral dip method (Clough and Bent, 1998). For GUS staining, seedlings, and different plant parts were collected from the T2 transgenic plants and stained with 1 mM X-Gluc in the buffer with 100 mM NaP0_4_ pH 7.0, 1 mM EDTA, 5 mM potassium ferrocyanide, 5 mM potassium ferricyanide, 0.1% Triton X-100 at 37°C overnight. The samples were washed three times with 70% ethanol at 60°C to remove chlorophyll and the GUS-staining images were taken under a dissection microscope.

### Subcellular Localization of *AtSOT12*-GFP Fusion Protein

The *AtSOT12* ORF was PCR-amplified by using the primer pairs, forward, acgcgtcgacatgtcatcatcatcatcagttcctgc (*SalI* site is underlined), and reverse, ataagaatgcggccgccaagaagaaaatttaagaccagaacctttaa (*NotI* site is underlined) and inserted in-frame into the Gateway entry vector pENTR1A. The *AtSOT12-GFP* was created through recombination between pENTR1A:*AtSOT12* and the destination vector pMDC43 using Gateway LR Clonase II Enzyme Mix (Invitrogen). The pMDC43-*AtSOT12-GFP* construct was sequenced to confirm the fusion sequences, and then transformed into *Arabidopsis* Col-0 wild type plants by floral dip method (Clough and Bent, 1998). The T2 transgenic lines were selected, and GFP images were taken by using an Olympus IX81 inverted laser scanning confocal microscope system.

### Bisulfite Sequencing

Ten-day-old seedlings of *Arabidopsis* Col-0 wild type grown on ½ MS agar medium with or without NaCl treatment were used for genomic DNA isolation. Genomic DNA was isolated using CTAB method. 2 μg of purified genomic DNA was used for bisulfite treatment using EpiTech Bisulfite kit (Qiagen) following the manual’s instruction. Thermal cycling conditions used for bisulfite conversion were as follow: 99°C for 5 min, 60°C for 25 min, 99°C for 5 min, 60°C for 85 min, 99°C for 5 min, 60°C for 175 min, and 20°C for overnight incubation. Bisulfite treated DNA was purified using clean-up column provided by the kit, and 5 μL of the treated DNA samples were used as template for PCR reaction with specific primers. The reverse primer (R1: tttcttatatcaaatcttcatctcccaa) was added to the reaction, and after the primer extension reaction (10 cycles of 95°C for 1 min, 60°C for 3 min, 72°C for 3 min), the forward primer (F1: ggttttgattttagatttttttgttaagaat) was added into the reaction mixture and the second PCR reaction (10 cycles of 95°C for 1 min, 60°C for 1.5 min, and 72°C for 2 min, and 30 cycles of 95°C for 1 min, 50°C for 1.5 min, and 72°C for 2 min, and one cycle of 72°C for 10 min) was followed. The PCR products were used for nested PCR to further enrich the amplified products. The primers used for nested PCR were: nested F2: ttttgttaagaatttgttttattaatttagtt and nested R2: ctcccaaataaacaaaaactaataataataataac. The PCR products from the nested PCR reaction were purified from agarose gel electrophoresis and then cloned into pGEM-T Easy Vector (Promega). Plasmid DNA from about 40 independent clones were isolated and sequenced using Big-Dye Terminator Cycle Sequencing Kit (Applied Biosystem), and ABI3100 Genetic Analyzer (Applied Biosystem). The methylated cytosine residues (CNN) which were not converted to thymine were calculated through alignment of the sequenced DNA sequences with the *AtSOT12* promoter sequence.

### 5′ and 3′ RACE PCR

RACE PCR was performed essentially following the previous described method by Jiang et al. (2013). Briefly, 10-day-old seedlings of *Arabidopsis* Col-0 wild type grown on ½ MS agar medium with or without NaCl treatment were used for total RNA isolation using Plant RNA Purification Reagent (Sigma). cDNA was generated from 2 μg of total RNA by reverse transcription PCR using AMV reverse transcriptase (Promega) and purified using MinElute PCR Purification Kit (Qiagen). For 5′ RACE, the cDNA was incubated with terminal transferase (New England Biolabs) and excess dATP to add poly-A to the 5′ end. The poly-A attached cDNA was then used as template for PCR amplification using the primer ctgatctagaggtaccggatcc-dT_(17)_ and *AtSOT12*-specific primer tcagactcttgtttcttgtgtcaga. The PCR products were used as template for a nested PCR using the adaptor primer ctgatctagaggtaccggatcc and a nested primer ttcatctcccaagtaagcaggaac. After the nested PCR reaction, final PCR products were cloned into pGEM-T Easy Vector (Promega) and 20–40 positive clones were isolated and sequenced using Big-Dye Terminator Cycle Sequencing Kit (Applied Biosystem), and ABI3100 Genetic Analyzer (Applied Biosystem). The addition of a G at the very end of the cDNA as an indication of a capped mRNA was counted from the sequenced cDNAs.

For 3′ RACE, the purified cDNA synthesized by reverse transcription with oligo dT_(17)_ was amplified by PCR using the adaptor-dT(17) and the *AtSOT12* specific primer ttgccaaatggaatagagactaaaac. A nested PCR was followed using the adaptor primer and an *AtSOT12* specific nested primer ggagagatactttgagtgagtcattgg. The final PCR products were cloned into pGEM-T Easy Vector (Promega) and plasmids from at least 30 independent colonies were sequenced. The polyadenylation sites were analyzed according to the sequences in conjunction with the poly-A.

## Results

### Salt Inducible Promoter Element Resides in the 5′-UTR of *AtSOT12* Gene

**FIGURE 1:**
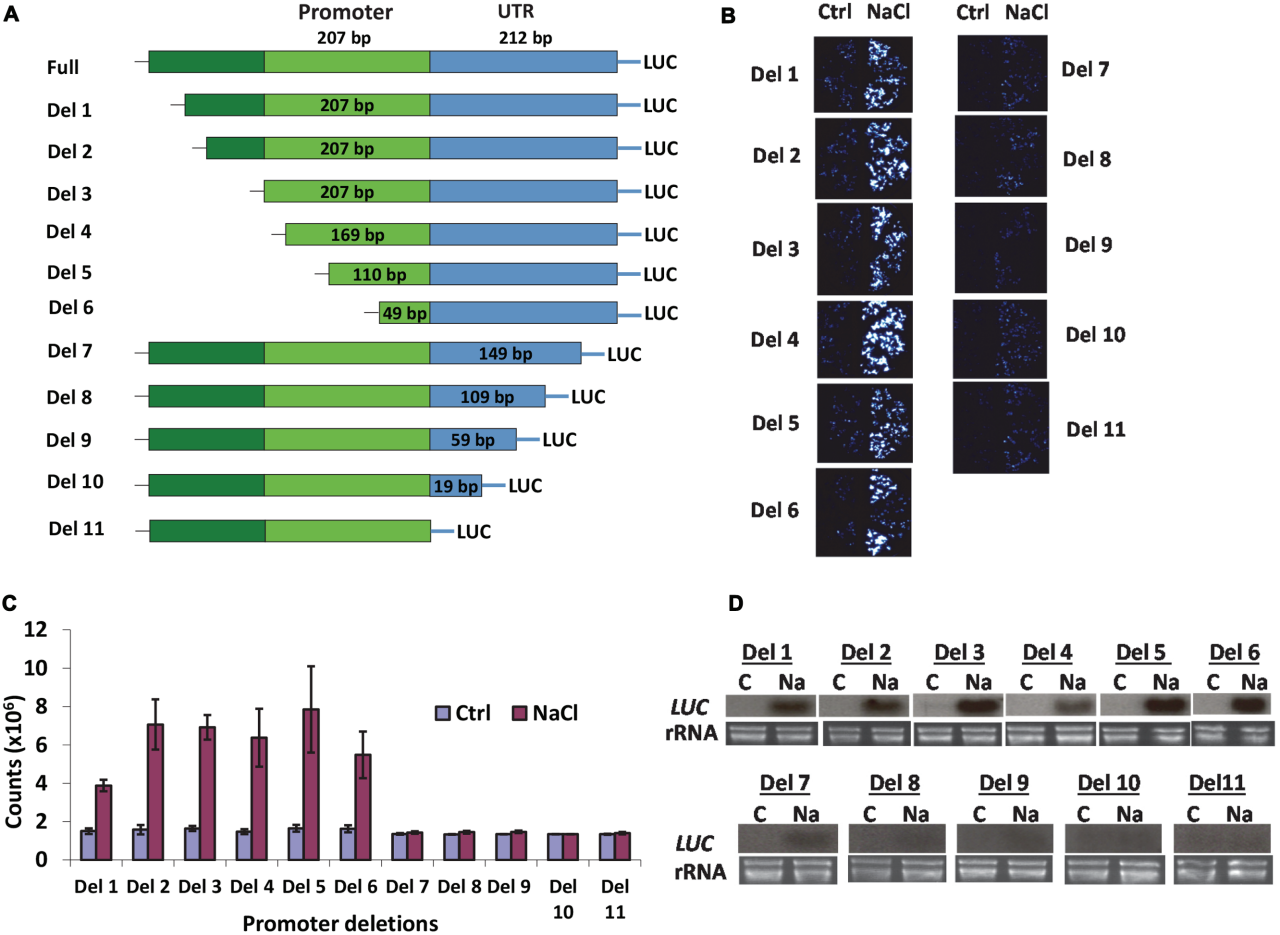
***AtSOT12* promoter analysis. (A)** A schematic showing deletion constructs used for studying promoter activity in transgenic *Arabidopsis*. **(B)** Luciferase imaging of the transgenic seedlings harboring each of the deletion constructs. Four independent transgenic lines were tested. Ctrl: control without NaCl treatment; NaCl: treated with 200 mM NaCl for 5 h. **(C)** Quantitative measurement of luciferase activity showing in **(B)**. Values are shown as mean ± SD (*n* = 10). **(D)** Northern blotting for detection of the luciferase transcripts levels in the seedlings harboring different deletion constructs. C: control; Na: 200 mM NaCl treatment for 5 h. rRNA is shown as loading controls.

Our previous research indicated that *AtSOT12* gene is a multiple stress inducible gene and can be highly induced by NaCl treatment (Baek et al., 2010; Chen et al., 2015). The approximately 1 kb sequence in front of the initiation codon (ATG) of the *AtSOT12* gene including 208 bp 5′-UTR, 207 bp promoter region, and 624 bp upstream sequence (**Figure 1A**) was validated to be a salt inducible promoter that could drive salt inducible expression of the luciferase reporter gene in *Arabidopsis* (Jiang et al., 2013). In this study, we attempted to locate the salt inducible element in the *AtSOT12* promoter. A series of deletions from both 5′ and 3′ end of the promoter were made (**Figure 1A**), and stable *Arabidopsis* transgenic plants harboring the promoter deletions driving luciferase expression were generated. Luciferase imaging (**Figure 1B**) and quantification (**Figure 1C**) showed that all 6 deletions from the 5′ end of the promoter did not affect the induced expression of the luciferase gene, while deletion from the 3′ end almost entirely abolished the induction of luciferase expression in response to NaCl treatment. Northern blotting analysis further confirmed these observations; all deletions from the 5′ end exhibited clear induced expression of luciferase gene by NaCl treatment, but the deletions from 3′ end diminished or abolished the induction (**Figure 1D**). The deletion 7 (Del-7) from the 3′ end still showed a low level of induction, while all other 3′ end deletions almost completely abolished the induction (**Figure 1D**). These results indicate that the DNA element responsible for the salt induced expression is within the ∼100 bp region in the 5′ UTR in conjunction with the coding region of *AtSOT12* gene.

### *AtSOT12* Expression Pattern and Subcellular Localization

**FIGURE 2:**
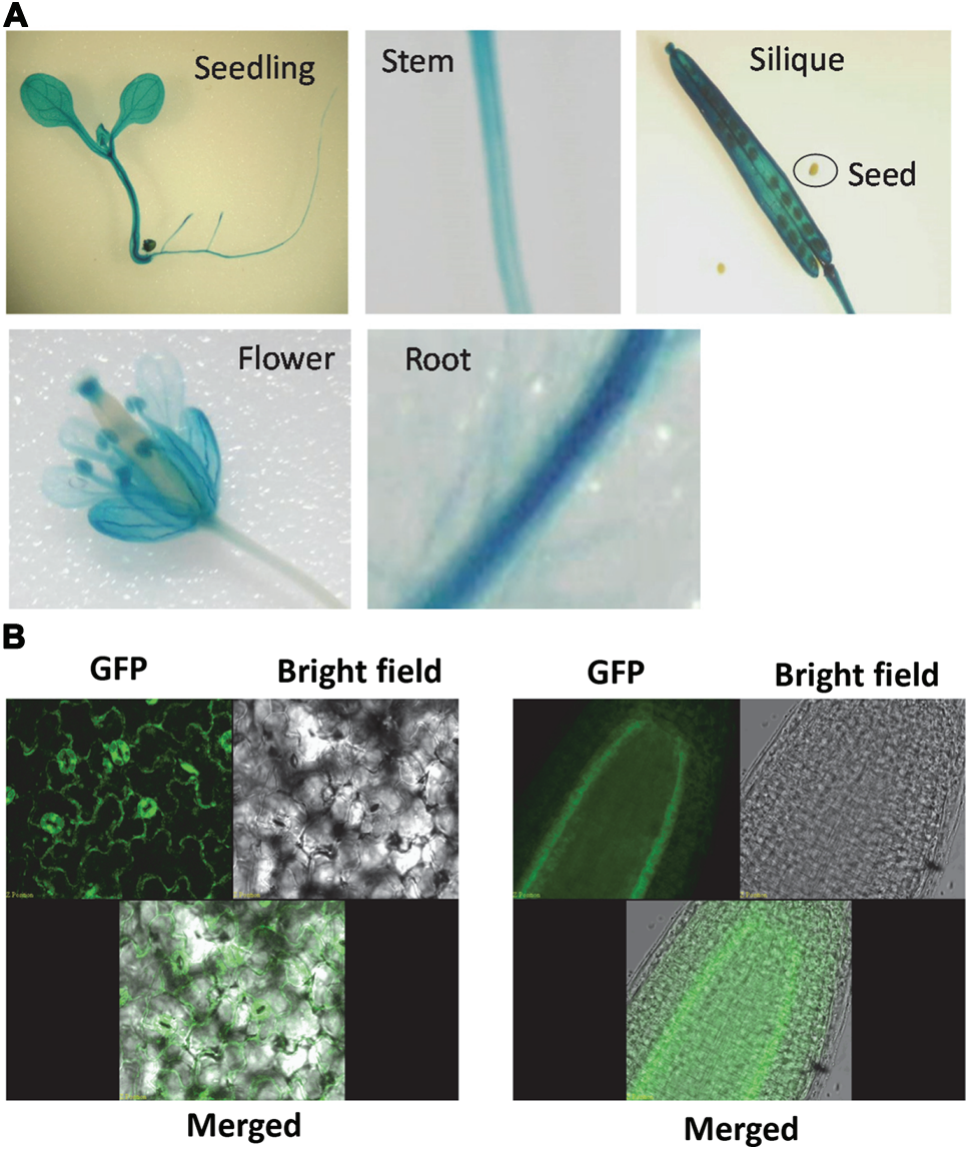
***AtSOT12* gene expression and protein localization. (A)** GUS staining of the transgenic plants harboring *AtSOT12* promoter-GUS fusion. **(B)** AtSOT12-GFP fusion protein localization visualized using Confocal microscope. Left panel, leaf GFP images; right panel, root GFP images.

Promoter-GUS analysis was used to assess the expression pattern of *AtSOT12* gene. As shown in **Figure 2A**, GUS expression was detected in most tissues and organs of the plants. In seedling, GUS expression was observed in leaf, hypocotyl, and root with stronger expression in the vascular tissues. In flower, the expression level of GUS was high in sepals, anthers and stigma, and weak in petals, style and ovary of the pistil. GUS expression was also detected in stem and silique tissues, but not in the seeds within the silique.

The subcellular localization of AtSOT12 was visualized by using AtSOT12-GFP fusion protein driven by the constitutive 35S promoter. In the transgenic plants harboring this fusion protein, the GFP was mainly localized in the cytosol, which was evidenced by the GFP signal being a thin layer along with the plasma membrane due to a central vacuole in the leave epidermal cells. The cytosolic localization was also observed in the guard cells and root pericycle cells in which vacuoles without GFP are identifiable (**Figure 2B**). Interestingly, the GFP signal was not uniform in the leaf and root cells. Stronger GFP was detected in the guard cells and the root pericycle cells. This suggests that AtSOT12 protein level may be modulated in a cell-specific manner via post-transcriptional and/or post-translational regulation.

### DNA Methylation in the Promoter of *AtSOT12* Gene

**FIGURE 3:**
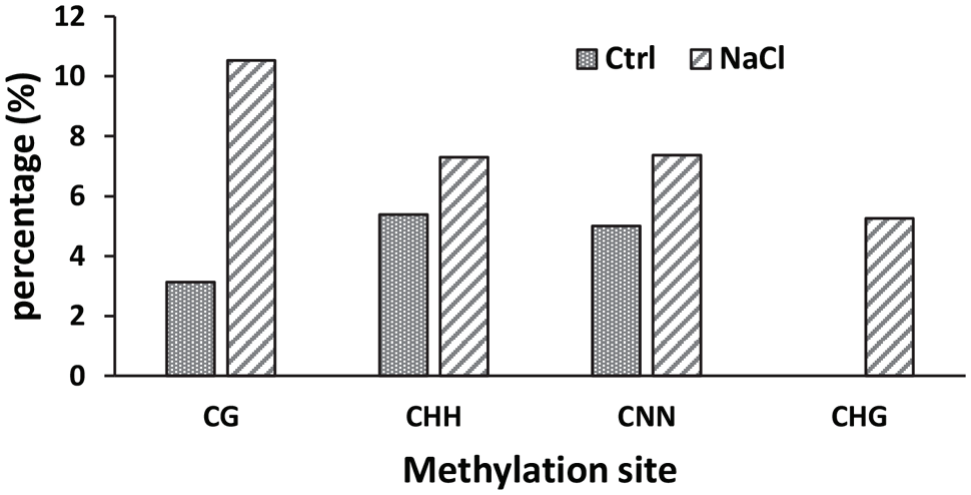
**Methylation of *AtSOT12* promoter region including 116 bp promoter sequence plus the 5′-UTR sequence with or without NaCl treatment**.

To determine whether promoter methylation contributes to the repression of *AtSOT12* at normal growth conditions or induced expression by salt stress, bisulfite sequencing was employed to analyze the methylation level of the promoter and the 5′ UTR. As shown in **Figure 3**, the total methylation of cytosine residues (CNN) was very low, 5.0% in the seedlings under normal growth conditions and 7.4% after NaCl treatment. CG methylation was 3.1% in the control seedlings but increased to 10.5% after salt stress treatment. Methylation of cytosine in the form of CHH and CHG was also slightly increased after salt stress treatment. Since DNA methylation generally represses gene expression, it is unlikely that the induced expression of *AtSOT12* by NaCl treatment is through changes in promoter methylation. Low level of cytosine methylation also suggest that promoter methylation may not contribute to the repression of *AtSOT12* expression under normal growth conditions.

### mRNA 5′ Capping and 3′ Polyadenylation Site Selection in Response to Salt Stress

**FIGURE 4:**
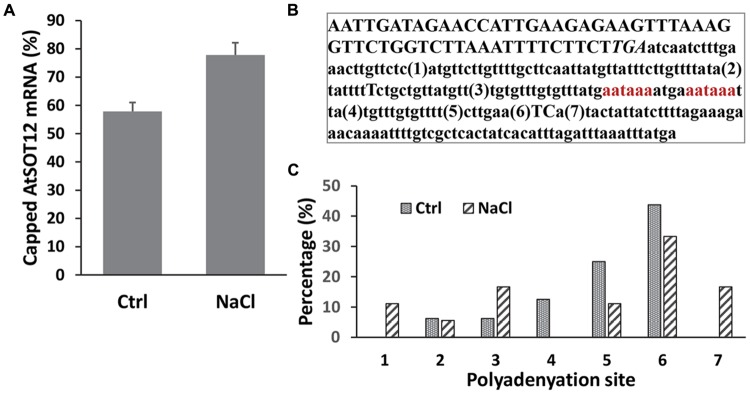
**5′ capping and polyadenylation site determination of *AtSOt12* gene transcripts in response to NaCl treatment. (A)** Percentage of capped mRNA of *AtSOT12* before and after NaCl treatment. Values are mean ± SD (*n* = 3). **(B)** Sequence at the 3′ end of the *AtSOT12* gene. Numbers in parenthesis are the polyadenylation sites detected. The stop coden TGA is italicized. The putative polyadenylation signal sequences aataaa are red-colored. **(C)** Percentage of *AtSOT12* transcripts polyadenylation at the indicated sites before and after NaCl treatment. Ctrl: control without NaCl treatment; NaCl: 200 mM NaCl treated for 5 h.

mRNA capping and polyadenylation are two crucial co-transcriptional processes in all eukaryotic genes. To determine whether salt stress affects these two processes during *AtSOT12* transcription, *AtSOT12* mRNA capping ratio and polyadenylation sites were analyzed. 5′ RACE was used to determine the capping ratio by analyzing the addition of an extra G residue at the very 5′ end of the cDNA, which was validated as a reliable method for capping analysis (Ohtake et al., 2004; Jiang et al., 2013). **Figure 4A** shows that NaCl treatment increased the capping of *AtSOT12* mRNA. Determination of polyadenylation sites revealed multiple sites for *AtSOT12* mRNA polyadenylation (**Figure 4B**). Although the major polyadenylation site (site 6 shown in **Figure 4B**) remained unchanged before and after salt treatment, other polyadenylation sites exhibited difference between control and salt treated seedlings. Salt stress caused more disperse distribution of the polyadenylation sites (**Figure 4C**). These results suggest that salt stress not only induces gene expression through the promoter, but also modulate posttranscriptional mRNA modifications which may be a part of gene upregulation mechanism.

### Reactive Oxygen Species Accumulation and Signaling are Required for Repression of *AtSOT12* Gene

**FIGURE 5:**
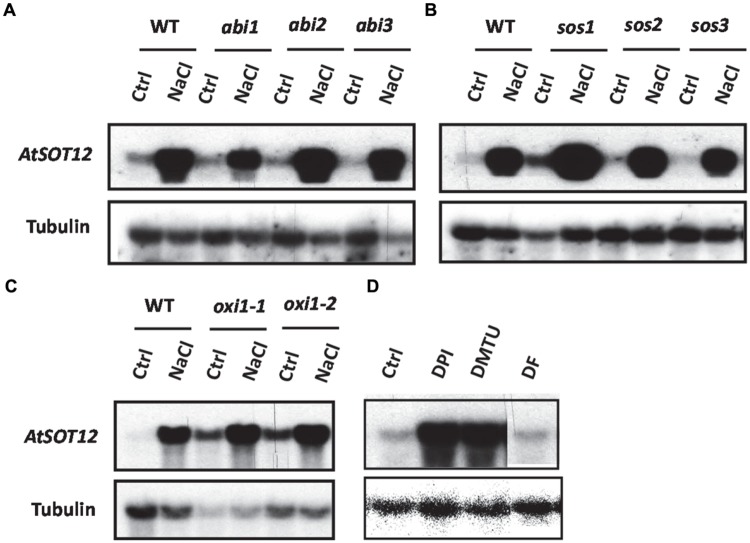
**Northern blotting showing *AtSOT12* transcript levels. (A)** *AtSOT12* transcript levels in wild type and *abi1-1, abi2-1*, and *abi3-1* mutants with or without NaCl treatment. **(B)** *AtSOT12* transcript levels in wild type and *sos1, sos2*, and *sos3* mutants with or without NaCl treatment. **(C)** *AtSOT12* transcript levels in wild type and *oxi1-1* (CS85631 from ABRC) and *oxi1-2* (CS85668 from ABRC) mutants with or without NaCl treatment. **(D)** *AtSOT12* transcript levels in wild type in response to DPI, DMTU, and DF treatment. Tubulin used as a loading control. Ctrl: control without NaCl treatment; NaCl: 200 mM NaCl for 5 h; DPI: 100 uM DPI for 3 h; DMTU: 15 mM DMTU for 3 h; DF: 1 mM DF for 3 h.

The transcript level of *AtSOT12* with or without salt stress was monitored in many genetic mutants to determine the signaling pathways controlling *AtSOT12* regulation. Among the mutants tested, *AtSOT12* expression in the three ABA insensitive mutants, *abi1, abi2*, and *abi3*, showed different response to NaCl treatment when compared with the wild type (**Figure 5A**). The *abi1* mutant displayed clearly diminished induction of *AtSOT12* expression, while the expression levels of *AtSOT12* in *abi2* and *abi3* are comparable with that in the wild type (**Figure 5A**). This result indicates that the induced expression of *AtSOT12* by NaCl is partially through ABI1-mediated signaling pathway. In contrast, the salt sensitive mutant *sos1* (Shi et al., 2000, 2002) displayed increased induction of *AtSOT12* expression by NaCl treatment, while *sos2*, and *sos3* showed the induction level similar with the wild type (**Figure 5B**). This suggests that more NaCl accumulation and severe damage by salt stress may be the primary cause of salt induced expression of *AtSOT12*. Under normal growth conditions, the *AtSOT12* expression level in the oxidative stress mutant *oxi1* (Rentel et al., 2004) was much higher than that in the wild type (**Figure 5C**), which indicates that ROS signaling may be required for the maintenance of the repression status of *AtSOT12* under normal growth conditions. To further support this notion, two ROS scavengers, dimethylthiourea (DMTU) and deferoxamine (DF), and the NADPH oxidase inhibitor diphenylene iodonium (DPI) were used to study the involvement of ROS in the repression of *AtSOT12* gene. DMTU is a potent ROS scavenger and DF is an iron chelator to prevent hydroxyl radical formation from H_2_O_2_. As shown in **Figure 5D**, DMTU treatment, but not DF treatment, strongly induced the transcript level of *AtSOT12*, which suggests that repression of *AtSOT12* may need specific forms of ROS. DPI treatment also strongly increased the transcript level of *AtSOT12*, suggesting that ROS production mediated by the plasma membrane-bound NADPH oxidase may be involved in the repression of *AtSOT12* gene.

## Discussion

A eukaryotic promoter often contains a core promoter and regulatory sequences that can be either upstream or downstream of the core promoter. These regulatory sequences provide binding sites for transcription factors that either enhance or prevent the binding of RNA polymerase onto the core promoter. The stress responsive *cis*-elements identified in plant promoters are often located upstream of the transcription start site (TSS). For example, the extensively studied ABA responsive element ABRE and drought responsive element DRE/CRT are in the promoter region between -51 and 450 in *Arabidopsis* (Yamaguchi-Shinozaki and Shinozaki, 2005). The DRE/CRT elements in the promoters of drought and COR genes in soybean are also found in the upstream sequence of TSS (Kidokoro et al., 2015). Sequence analysis of the promoters of cold- and dehydration-inducible genes in *Arabidopsis*, rice and soybean revealed that the ABRE and DRE elements are overrepresented in the promoter region between -51 and -100 (Maruyama et al., 2012). Identification of the heat responsive promoter elements also indicated that the HSEs are often located in the promoter between -50 and -200 (Guan et al., 2010; Kurdrid et al., 2010; Mittal et al., 2011; Yoshida et al., 2011). However, our promoter analysis revealed a *cis*-element that is required for salt inducible expression of the reporter gene and located downstream of the TSS. The ∼100 bp region in the 5′ UTR linking the coding sequence of the *AtSOT12* gene was identified to be important for gene upregulation by salt stress (**Figure 1**). A putative DRE element located ∼150 bp from the 3′ end of the UTR was predicted using the online program PLACE (https://sogo.dna.affrc.go.jp/cgi-bin/sogo.cgi?sid=&lang=en&pj=640&action=page&page=newplace). In addition, many other putative *cis*-elements in the 5′ UTR were also identified by this online tool. Whether the putative DRE in combination with other *cis*-elements in the 5′ UTR are responsible for the salt induced expression requires further research. Nevertheless, the identification of a salt responsive *cis*-element downstream of the TSS may lead to identifying novel regulatory mechanisms of salt responsive gene expression. One possible role of the 5′ UTR sequence in the *AtSOT12* gene is to provide histone modification sites that regulate the chromatin status in response to stress conditions. It was shown that the gene activation marker H3K4me3 changes dramatically in the dehydration responsive genes, and this modification is predominantly present at the 5′-ends of most transcribed genes (van Dijk et al., 2010). It is possible that the 5′ UTR sequence of *AtSOT12* genes is a hotspot for H3K4me3 modification to de-repress the gene, which might be a pre-requisite for activation of *AtSOT12* gene expression by salt stress.

Many genes are kept inactive at normal growth conditions but activated in response to environmental cues. In contrast to extensive studies on the gene induction mechanisms involving transcription factors and *cis*-acting elements, the mechanisms of repression of stress inducible genes is still poorly understood. DNA methylation is one of the gene silencing mechanisms, but it seems not to be the mechanism for *AtSOT12* gene repression. This is supported by our analysis of cytosine methylation revealing a very low methylation in the promoter and 5′ UTR of *AtSOT12* gene and a slight increase in cytosine methylation after salt stress treatment (**Figure 3**). Repression of *AtSOT12* gene is likely to be an active process involving protein complex including histone deacetylase. A mutant screening for altered expression of the luciferase reporter gene driven by the *AtSOT12* promoter (with 5′ UTR) identified a mutation in a histone deacetylase that caused elevated expression of luciferase gene under normal growth conditions (Shi lab unpublished data). This suggests that histone deacetylation in the *AtSOT12* promoter and 5′ UTR is important for its repression. The repression complex may also include components such as SHI1 and FRY2 that were also identified in our mutant screening. SHI and FRY2 repress *AtSOT12* gene partly by decreasing mRNA capping (Jiang et al., 2013). In this study, we also found that mRNA capping of *AtSOT12* gene is increased after salt stress treatment (**Figure 4A**). This suggests that salt stress may cause dissociation of the repressor complex including SHI1 and FRY2 thus increase mRNA capping. Salt stress also altered the polyadenylation sites of the *AtSOT12* mRNA (**Figure 4C**), which may be resulted from the transcription complexes that include different components for very low level of *AtSOT12* transcription under normal growth condition and high level transcription under salt stress conditions.

How the repression state of stress inducible genes is maintained under normal growth conditions is another interesting question. Our results suggest that physiological level of ROS and ROS signaling might be involved in the repression of *AtSOT12* gene at normal growth conditions. The basal expression level of *AtSOT12* in the oxidative signaling mutant *oxi1* is elevated significantly (**Figure 5C**), suggesting that ROS signaling is required for *AtSOT12* gene repression. OXI1 is a serine/threonine protein kinase that mediates active oxygen species signaling by activating downstream MAPK kinase cascade and other downstream responses (Rentel et al., 2004). Plants and other living organisms constantly produce ROS in the cells as a result of the most essential biochemical processes such as photosynthesis and respiration, while plant cells also generate apoplastic ROS through the action of plasma membrane-bound NADPH oxidase (Torres and Dangl, 2005). NADPH oxidase converts O_2_ into superoxide anion (O_2_^-^) which is dismutated into H_2_O_2_. H_2_O_2_ can generate hydroxyl radical (OH^-^) in the present of transition metals such as Fe (Sagi and Fluhr, 2006; Kurusu et al., 2015). Under normal growth conditions, ROS homeostasis is maintained through the balance of production and removal, and the physiological level of ROS may serve as an important signal mediating normal levels of gene expression including repression of the stress inducible genes. Stress conditions including salt stress result in increased production of ROS thus reset the cellular redox status, which may signal to a battery of downstream components such as protein phosphatases and HDACs that trigger changes in chromatin structure. ROS and redox signaling have been implicated in numerous molecular response including histone acetylation, gene transcription, mRNA stability, etc. (Forman and Torres, 2002; Temple et al., 2005; Chung et al., 2008; Miller et al., 2010; Escobar et al., 2012). Our results suggest that the *AtSOT12* repression under normal growth conditions requires ROS and ROS signaling, and the change in redox status after salt stress may be a signal for de-repression of the *AtSOT12* gene through, for example, histone modifications and chromatin remodeling, which could be the prerequisite for the gene activation via the binding of the activator to the *cis* promoter elements. DPI treatment increased the expression of *AtSOT12* (**Figure 5D**), indicating that NADPH oxidase may be the source of ROS production and ROS signaling for *AtSOT12* repression. DMTU but not DF treatment elevated the expression of *AtSOT12* (**Figure 5D**), which suggests that physiological levels of O_2_^-^ and/or H_2_O_2_ but not OH^-^ radical may be the signaling ROS for maintaining repression status of *AtSOT12* gene.

In addition to the transcriptional regulation of *AtSOT12*, posttranscriptional processes may also be involved in modulating AtSOT12 protein abundance. Our study on AtSOT12-GFP localization revealed a cell-type preferential accumulation of AtSOT12 protein. Although its expression is controlled by the constitutive 35S promoter, the AtSOT12-GFP fusion proteins predominantly accumulated in the guard cells in the leaves and the pericycle cells in the roots. This suggests that AtSOT12 proteins are modulated at posttranscriptional levels in these cell types through the control of mRNA stability, protein translation, or protein stability. Although we could not pinpoint which posttranscriptional step modulating AtSOT12 abundance without further research, this result indicated a possible important role of AtSOT12 in these cell types. The guard cells control stomatal movement in response to environmental changes and ABA plays crucial roles in this process. It has been shown that sulfate availability influences ABA under salt stress (Cao et al., 2014). Perhaps AtSOT12 is one of the key enzymes regulating sulfur pools in the guard cells due to its strong induction by salt stress and its wide substrates selectivity (Baek et al., 2010; Chen et al., 2015). Through modulating sulfur contents, AtSOT12 may regulate ABA levels thus play a role in guard cell function. Alternatively, sulfonation of small molecules including hormones such as salicylic acid and brassinosteroids by AtSOT12 sufotransferase (Baek et al., 2010) may be a molecular response of the guard cells to stress conditions such as salt stress. Predominant accumulation of AtSOT12 in the pericycle cells in roots may indicate a role of AtSOT12 in sulfur assimilation in response to salt stress. Salt stress has been reported to increase sulfur contents and sulfur assimilation (Rodríguez-Hernández et al., 2014; Nazar et al., 2015). Thus, it is conceivable that the pericycle cells are sites for sulfur accumulation, transport, and assimilation and AtSOT12 functions in these cells for sulfur assimilation by sulfonating a variety of small molecules (Chen et al., 2015). Sulfur assimilation via AtSOT12 in the pericycle cells may be a molecular response contributing to salt stress tolerance.

## Author Contributions

JC, BW, J-SC, HC performed the experiments described in this paper. JC, CL, YR, HS were involved in organizing the data and drafting the manuscript. HS designed the experiments and extensively involved in the writing and finalizing the manuscript.

## Conflict of Interest Statement

The authors declare that the research was conducted in the absence of any commercial or financial relationships that could be construed as a potential conflict of interest.
