# Supplementary material for: The role of promoter cis-element, mRNA capping, and ROS in the repression and salt-inducible expression of AtSOT12 in Arabidopsis
Source: Front Plant Sci. 2015 Nov 6;6:974. doi: 10.3389/fpls.2015.00974 (PMC4635225; doi:10.3389/fpls.2015.00974)
Supplement: Supplementary file 1 [file Table_1.DOCX]

**Supplemental Table 1.** List of primers for the deletion constructs in this study

_______________________________________________________________________

Primer Name Primer Sequence (5’ to 3’)

Full-F CCCCCCGGGGAAGGTTTCCACCTTCACACTC

Full-R AAAACTGCAGTGTTGAGACTTGAGAGATCGATCA

Del 1-F ATCGCCCGGGGCCGAGGGTCTAGTATATACG

Del 2-F ATCGCCCGGGGAATGGAATGGAGACTAAGGC

Del 3-F ATCGCCCGGGGAATCTGCTTTACCAACTTAG

Del 4-F ATCGCCCGGGGAGCTTTTTTTTTTCTTCTTTTTAT

Del 5-F ATCGCCCGGGGACCAAAGTCACCAAACTGGTC

Del 6-F ATCGCCCGGGAAGAGTCGTGATTCTCCTTC

Del 7-R ATCGCTGCAGTATGTATCTATGATGCTAATGC

Del 8-R ATCGCTGCAGGAATGAGAAGGTTGTTCTAC

Del 9-R ATCGCTGCAGGATATTAGTTTGGGTGGTGAA

Del 10-R ATCGCTGCAGCTTGAAGAGCACGTAGAATA

Del 11-R ATCGCTGCAGATAATATTTAAAGATAACAA

_______________________________________________________________________
